# Association between *PLA2G12A* Polymorphisms and Schizophrenia in a Han Chinese Population from Northeast China

**DOI:** 10.1371/journal.pone.0159584

**Published:** 2016-07-19

**Authors:** Guang Yang, Hongqin Xu, Huiping Zhang, Qiong Yu, Yanhua Wu, Jieping Shi, Wenwang Rao, Yueyue You, Changgui Kou, Yaqin Yu

**Affiliations:** 1 Department of Epidemiology and Biostatistics, School of Public Health, Jilin University, 1163 Xinmin Street, Changchun, 130021, Jilin province, China; 2 Department of Hepatology, First Hospital of Jilin University, Jilin University, Changchun, Jilin, 130021, China; 3 Department of Psychiatry, Yale University School of Medicine, New Haven, United States of America; 4 VA Medical Center, VA Connecticut Healthcare System, West Haven, United States of America; 5 Division of Clinical Epidemiology, First Hospital of Jilin University, Changchun, Jilin, 130021, China; National Cancer Center, JAPAN

## Abstract

**Objective:**

The purpose of this study was to explore the association between single nucleotide polymorphisms (SNPs) in the phospholipase A2 (PLA2), group XIIA gene (*PLA2G12A*) and schizophrenia.

**Methods:**

This study included 1,063 schizophrenia patients and 1,103 healthy controls from a Han Chinese Population in Northeast China. Four tagSNPs (rs11728699 in intron 1, synonymous rs2285714 in exon 3, rs3087494 in the 3’ UTR, and rs7694620 in the downstream region) in *PLA2G12A* were selected, and they were genotyped by the MALDI-TOF-MS technology. The Chi-square (χ^2^) test and haplotype analysis were performed to analyze the association of *PLA2G12A* SNPs and schizophrenia using the software packages SPSS 16.0 and Haploview 4.2.

**Results:**

Among the four tagSNPs, only SNP rs3087494 in the 3’ UTR of *PLA2G12A* showed significant differences in both allele frequencies (χ^2^ = 20.136, *P*<0.001) compared to healthy controls. The minor allele G of SNP rs3087494 is potentially a predictive factor for schizophrenia (*OR* = 0.753, 95% *CI*: 0.665–0.882). The frequency distribution of haplotypes consisting of specific alleles of two SNPs (rs7694620-rs3087494 or rs3087494-rs2285714), three SNPs (rs7694620-rs3087494-rs2285714 or rs3087494-rs2285714-rs11728699), or all four SNPs (rs7694620-rs3087494-rs2285714-rs11728699) was significantly different between schizophrenia patients and control subjects (*P*<0.001).

**Conclusions:**

Our study demonstrated that *PLA2G12A* SNPs or haplotypes might influence the susceptibility to schizophrenia in the Han Chinese population from Northeast China.

## Introduction

Schizophrenia is a severe and complex psychiatric disorder that is characterized by positive symptoms (such as hallucinations and delusions) and negative symptoms (such as lack of motivation and interest, social withdrawal, and flattened affect) [1]. Schizophrenia is a common disease, with a lifetime prevalence of approximately 1% [2]. This disease has a marked impact on the quality of life and causes a large social and economic burden. Schizophrenia accounts for approximately 2.8% of the global burden of diseases, according to the statistics published by the World Health Organization (WHO) in 2001. Previous studies have consistently demonstrated that the pathogenesis of schizophrenia is closely related to genetic and environmental factors [3, 4]. The heritability of schizophrenia is estimated to be 64–80%, but the genetic basis of schizophrenia is still far from clear because of the lack of specific biological markers. The effect of genetic factors on schizophrenia risk has been demonstrated by a number of family and twin studies. Genome wide association studies (GWAS) have identified a set of common genetic variants predisposing an individual to schizophrenia [5, 6].

Previous studies have reported abnormal metabolic processes in the membrane phospholipids among patients with schizophrenia [7, 8]. Phospholipase A2 (PLA2) is an important enzyme that catalyzes the hydrolysis of the sn-2 acyl bond of phospholipids to release arachidonic acid and lysophospholipids. Gattaz et al [9] first reported an increased PLA2 activity in the serum, plasma, and platelets of psychiatric patients without drug treatment, and several follow-up studies confirmed this finding [7, 10]. Recent studies by Smesny [11] demonstrated the PLA2 activity was significantly associated with brain structure changes in schizophrenia patients. Eckert et al [12] found that the elevated PLA2 activity in the prefrontal cortex caused increased brain cell membrane fluidity in schizophrenia patients. These results indicated that PLA2 played a key role in the pathogenesis of schizophrenia. Numerous studies have shown that schizophrenia is related to activity changes in cytosolic PLA2 [13–15]. However, inconsistent findings on the association of schizophrenia and PLA2 gene polymorphisms were observed, and there were also studies indicating that schizophrenia is not influenced by the activity of cytosolic PLA2 [16–19].

In our previous studies, we found significant associations between PLA2 family gene polymorphisms and schizophrenia, including different clinical symptoms of schizophrenia. For instance, SNP rs1549637 in *PLA2G4C*, BanI SNP in the *PLA2G4A* locus, and SNP rs1648833 in *PLA2G4B* may contribute to the risk of schizophrenia [18]. To extend our previous findings, we further investigated the genetic association between *PLA2G12A* polymorphisms and schizophrenia, and significant results were obtained.

## Materials and Methods

### Study population

We recruited 1,063 patients with schizophrenia from the Sixth Hospital of Changchun and 1,103 healthy controls from the First Hospital of Jilin University through a community-based study of the Han Chinese population in the Jilin Province of Northeast China. The patients were diagnosed independently by at least two experienced psychiatrists according to the diagnostic criteria of the International Statistical Classification of Diseases and Related Health Problems, Tenth Revision (ICD-10). The healthy control subjects were matched to the case subjects by gender and did not have a positive family history of schizophrenia or other mental disorders. All subjects gave written informed consent before participating in this study, which was approved by the Ethics Committee of the School of Public Health, Jilin University. Blood samples were obtained from participants. Demographic information on gender, age, ethnicity, family history and other measures were collected using a structural questionnaire.

### DNA extraction and SNP selection

Subjects donated 5 mL of blood for biochemical analysis. Genomic DNA from peripheral blood lymphocytes was extracted using the DNA Extraction Kit (Kangwei Biotech Company, Beijing, China). We selected four tagSNPs in *PLA2G12A* (rs11728699 in intron 1, synonymous rs2285714 in exon 3, rs3087494 in the 3’ UTR, and rs7694620 in the downstream region) based on linkage disequilibrium (LD) among SNPs in *PLA2G12A* analyzed using the Haploview 4.2 software (http://hapmap.ncbi.nlm.nih.gov/). The four SNPs we selected also adhered to the following standards: 1) the minor allele frequency of these SNPs was over 10% in the Chinese population with r^2^>0.8 and D^′^ = 1, 2) the SNPs were related to the functional involvement in the pathogenesis of schizophrenia, and 3) the SNPs were reported to have associations with other related disorders in previous studies. SNP genotyping was performed using matrix-assisted laser desorption ionization-time of flight mass spectrometry (MALDI-TOF-MS).

### Statistical analyses

Statistical analyses were conducted using the SPSS (Version 16.0, IBM SPSS, IBM Corp, Armonk, NY, USA). The Chi-square (χ^2^) test was used to analyze whether there were significant differences in gender distributions between cases and controls. Student’s t-test was used to analyze whether there were significant differences in age between cases and controls. For each SNP, a Hardy-Weinberg equilibrium (HWE) test was conducted in both case and control groups. The Chi-square (χ^2^) test was also used to compare SNP allele frequencies and genotype distributions between cases and controls. *P* values less than 0.05 were considered to be statistically significant. The Bonferroni correction was used to adjust for multiple comparisons.

## Results

### Subject characteristics

Our study included 2,166 subjects, comprised of 1,063 schizophrenia patients [589 males and 474 females; mean age (±S.D.), 34±12 years] and 1,103 healthy controls [566 males and 537 females; mean age (±S.D.), 36±10 years]. There was no significant difference in the gender distribution between cases and controls (χ^2^ = 3.65, *P* = 0.056). The genotype distributions of the four tagSNPs in the control group were not deviated from the Hardy-Weinberg equilibrium (*P*>0.05) (Table 1).

**Table 1:** The Hardy-Weinberg equilibrium results by goodness-of-fit Chi-square tests.

tag SNPs	Cases				Controls			
	H_o_	H_e_	*χ*^*2*^	*P*	H_o_	H_e_	*χ*^*2*^	*P*
rs7694620	0.443	0.492	10.37	0.001	0.472	0.492	1.88	0.171
rs11728699	0.456	0.455	0.01	0.942	0.428	0.453	3.18	0.075
rs2285714	0.343	0.343	0.00	0.987	0.322	0.341	3.57	0.059
rs3087494	0.382	0.489	45.44	<0.001	0.495	0.500	0.11	0.739

H_o_: observed heterozygosity; H_e_: expected heterozygosity; df = 1

### The genotype distribution and allele frequency of PLA2G12A tag SNPs

Genotype distributions and allele frequencies of the four tagSNPs in *PLA2G12A* are shown in Table 2. SNP rs3087494 in the 3’ UTR of *PLA2G12A* was significantly associated with schizophrenia. The genotype distribution and allele frequency of this SNP were significantly different between cases and controls (genotype distribution comparison: χ^2^ = 40.43, df = 2, *P* < 0.001; allele frequency comparison: χ^2^ = 20.14, df = 1, *P* < 0.001). Nevertheless, the three other SNPs (rs7694620, rs11728699 and rs2285714) in *PLA2G12A* were not significantly associated with schizophrenia by either genotype distribution (using additive or dominant models) or allele frequency comparisons between cases and controls.

**Table 2:** Genotype distribution and allele frequency differences between schizophrenia patients and healthy controls for four *PLA2G12A* SNPs.

SNPs	Genotype/Allele		Cases(%)	Controls(%)	*χ*^*2*^	*P*^a^	OR	95%CI	
								Lower	Upper	
rs7694620	Genotype	TT	357(34.16)	355(32.54)	1.82	0.403	1.000	—	—
		TC	463(44.31)	515(47.20)			0.894	0.737	1.085
		CC	225(21.53)	221(20.26)			1.012	0.799	1.283
		TC+CC (*vs*TT)	688(65.84)	736(67.46)	0.56	0.454	0.923	0.771	1.105
	Allele	T	1177(56.32)	1225(56.14)	0.01	0.908	1.000	—	—
		C	913(43.68)	957(43.86)			0.993	0.880	1.121
rs11728699	Genotype	TT	128(12.25)	145(13.22)	1.76	0.414	1.000	—	—
		TG	477(45.65)	470(42.84)			1.150	0.878	1.506
		GG	440(42.10)	482(43.94)			1.034	0.789	1.355
		TG+GG(*vs*TT)	917(87.75)	952(86.78)	0.45	0.501	1.091	0.846	1.407
	Allele	T	733(35.07)	760(34.64)	0.09	0.767	1.000	—	—
		G	1357(64.93)	1434(65.36)			0.981	0.865	1.113
rs2285714	Genotype	CC	644(60.87)	684(62.13)	1.73	0.422	1.000	—	—
		TC	363(34.31)	354(32.15)			0.918	0.766	1.101
		TT	51(4.82)	63(5.72)			1.163	0.792	1.708
		TC+TT(*vs*CC)	414(39.13)	417(37.87)	0.36	0.549	0.948	0.797	1.128
	Allele	C	1651(78.02)	1722(78.20)	0.02	0.888	1.000	—	—
		T	465(17.25)	480(21.80)			0.990	0.857	1.143
rs3087494	Genotype	AA	224(23.33)	264(24.74)	40.43	**<0.001**	1.000	—	—
		AG	367(38.23)	528(49.48)			1.221	0.977	1.525
		GG	369(38.44)	275(25.78)			0.632	0.499	0.801
		AG+GG(*vs*AA)	736(76.67)	803(75.26)	0.55	0.459	0.926	0.755	1.135
	Allele	A	815(42.45)	1056(49.48)	20.14	**<0.001**	1.000	—	—
		G	1105(57.55)	1078(50.52)			0.753	0.665	0.882

^a^ Significant *P* values (<0.00125, Bonferroni corrected α) are in bold.

OR = odds ratio; CI = confidence interval.

The four tagSNPs were not located in one haplotype block by linkage disequilibrium (LD) analysis (Table 3). There exists LD between adjacent sites. The four tagSNPs in *PLA2G12A* formed several haplotype systems. The frequency distributions of the haplotype systems (rs7694620-rs3087494, rs3087494-rs2285714, rs7694620-rs3087494-rs2285714, rs3087494-rs2285714-rs11728699, and rs7694620-rs3087494-rs2285714-rs11728699) were significantly different between cases and controls (*P*<0.001) (Table 4).

**Table 3:** Linkage disequilibrium (LD) of SNPs in *PLA2G12A*.

	Distance	Cases		Controls	
	(bp)	D’	r^2^	D’	r^2^
rs7694620-rs3087494	3,302	0.779	0.601	1.00	0.771
rs3087494-rs2285714	7,189	0.992	0.205	1.00	0.275
rs2285714-rs11728699	11,081	0.988	0.507	0.989	0.518

**Table 4:** The association between *PLA2G12A* haplotypes and schizophrenia.

Haplotypes	*Global χ*^*2*^	*P*
rs7694620-rs3087494	103.641	<0.001
rs3087494-rs2285714	24.831	<0.001
rs2285714- rs11728699	0.176	0.916
rs7694620-rs3087494- rs2285714	93.734	<0.001
rs3087494-rs2285714-rs11728699	25.050	<0.001
rs7694620-rs3087494-rs2285714-rs11728699	90.070	<0.001

### Gender-specific associations with the four variants

To investigate whether gender would play a role in the SNP association, we analyzed the statistics in males and females, respectively (Table 5). Furthermore, we found that the frequency distributions of genotypes and alleles at rs3087494 were significantly different between cases and controls in males (P<0.001), but not in females.

**Table 5:** Sex-specific genotype distribution and allele frequency differences between schizophrenia patients and healthy controls for four PLA2G12A SNPs.

Markers and sex		Genotype (%)			Model	OR(95% CI)	*P*^a^ value	Allele		OR(95% CI)	*P*^a^ value
rs7694620		TT	TC	CC				T	C		
M	Case	197	270	109	Dom	1.088(0.811–1.459)	0.573	664	488		
	Control	163	282	113	Rec	0.794(0.618–1.020)	0.071	608	508	1.137(0.963–1.342)	0.130
F	Case	160	193	116	Dom	0.773(0.574–1.041)	0.090	513	425		
	Control	192	233	108	Rec	0.920(0.709–1.193)	0.528	617	449	0.878(0.736–1.048)	0.151
rs11728699		TT	TG	GG				T	G		
M	Case	81	261	239	Dom	1.130(0.894–1.429)	0.306	423	739		
	Control	72	242	248	Rec	1.103(0.784–1.551)	0.575	386	738	1.094(0.922–1.299)	0.303
F	Case	47	216	201	Dom	1.017(0.792–1.307)	0.894	310	618		
	Control	73	228	234	Rec	0.713(0.483–1.053)	0.088	374	696	0.933(0.775–1.124)	0.467
rs2285714		CC	TC	TT				C	T		
M	Case	354	197	35	Dom	1.010(0.621–1.643)	0.968	905	267		
	Control	359	171	34	Rec	0.871(0.686–1.106)	0.257	889	239	0.911(0.748–1.110)	0.356
F	Case	290	166	16	Dom	1.627(0.872–3.035)	0.123	746	198		
	Control	325	183	29	Rec	1.039(0.807–1.339)	0.765	833	241	1.090(0.881–1.348)	0.426
rs3087494		AA	AG	GG				A	G		
M	Case	121	204	218	Dom	0.442(0.339–0.575)	**0.000**	446	640		
	Control	143	279	125	Rec	0.810(0.614–1.070)	0.137	565	529	0.652(0.551–0.773)	**0.000**
F	Case	103	163	151	Dom	0.979(0.738–1.298)	0.881	369	465		
	Control	121	249	150	Rec	1.082(0.800–1.462)	0.610	491	549	0.887(0.739-.065)	0.200

^a^ Significant *P* values are in bold.

OR = odds ratio; M = males; F = females; CI = confidence interval; Dom = dominant model; Rec = recessive model.

### Analysis of the interaction between gender and polymorphism

In the analysis of the interaction between polymorphism and gender, we found that only rs3087494 had an interaction with gender (P<0.05) and this interaction was negative (OR_EG_ = 0.426). (Table 6)

**Table 6:** Logistics regression of the interaction between SNPs in PLA2G12A.and gender.

SNP	Factor	*χ*^*2*^	P	OR	95%CI	
					Lower	Upper
rs7694620	G	2.939	0.086	1.262	0.967	1.647
	E	1.255	0.263	0.865	0.671	1.115
	EG	1.307	0.253	0.797	0.540	1.176
	Constant	0.261	0.610	0.957		
rs11728699	G	0.052	0.819	0.959	0.667	1.377
	E	5.089	0.024	0.572	0.352	0.929
	EG	2.348	0.125	1.535	0.887	2.655
	Constant	0.529	0.467	1.125		
rs2285714	G	0.193	0.661	0.762	0.226	2.567
	E	2.510	0.113	0.536	0.248	1.159
	EG	0.834	0.361	1.469	0.644	3.354
	Constant	1.295	0.255	1.921		
rs3087494	G	15.885	0.000	3.700	1.944	7.039
	E	0.656	0.418	0.895	0.685	1.170
	EG	4.328	0.037	0.645	0.426	0.975
	Constant	0.930	0.335	0.817		

## Discussion

In the present study, we conducted a case-control study to examine the effect of four *PLA2G12A* SNPs on schizophrenia risk in a Han Chinese population. We found that the 3’ UTR SNP rs3087494 was significantly associated with schizophrenia in our sample. In this association study, we found that the G allele of the rs3087494 SNP was associated with a decreased risk of schizophrenia, and this SNP was also detected to confirm its association with schizophrenia in males. Nevertheless, three other *PLA2G12A* SNPs (rs2285714, rs11728699, and rs7694620) did not show significant associations with schizophrenia. To the best of our knowledge, this is the first report of a significant association of the *PLA2G12A* SNP rs3087494 with schizophrenia. This finding supports the hypothesis that the *PLA2G12A* gene may represent a novel susceptibility gene for schizophrenia.

The PLA2G12A gene located on 4q25 encodes a member of the PLA2 gene family. PLAs are important signaling molecules and are related to various pathologies, such as tissue injury, inflammation and Alzheimer’s disease [20–22]. Furthermore, PLAs have been shown to be closely related to the etiology and pathogenesis of schizophrenia. In line with previous studies, the activity of PLA2 in serum was increased in drug-free schizophrenia and the enzyme activity was decreased after antipsychotic drug treatment [8].

The positioning of susceptibility genes for schizophrenia is one of the key methods of researching its etiology. The increased activity of PLA2 is the result of abnormal phospholipid metabolism [23]. The studies of candidate genes and genome scanning have acquired many positive results. However, these results are poorly replicated and have caused much dispute. One possible explanation is that PLA2 family gene variants only play a minor role in schizophrenia and only high-efficiency statistical analysis or joint analysis of genetic markers could discover their genuine functions. Another possible explanation is that SNPs associated with schizophrenia act only as markers of a real disease locus that is linked to the identified SNPs.

Due to the diversity in regions of linkage disequilibrium in the population, the reproducibility of relevant schizophrenia candidate genes is deficient in different populations. It has been reported that the activity of secretory PLA2 is increased in the blood and the brain tissue of patients with schizophrenia. PLA2 may be related to the pathogenesis of schizophrenia. The PLA2 gene family may contain susceptibility genes for schizophrenia or could be linked with a susceptibility gene.

Genetic association studies of diseases based on individual SNP data analysis may not able to draw a definite conclusion because the information provided by a single SNP is incomplete. Negative results from a specific SNP cannot rule out the possible association of diseases with other surrounding SNPs. Additionally, a positive association between a SNP and a disease does not necessarily indicate a significant impact of that SNP on disease risk because the identified SNP may be in linkage disequilibrium (LD) with a real disease locus that has not been discovered yet. Therefore, we can carry out a more in-depth investigation by performing haplotype analysis. Haplotypes are comprised of a collection of alleles of two or more tightly linked loci on a chromosome, and haplotype blocks are formed by two or more tightly linked SNPs.

Our study investigated whether the four *PLA2G12A* SNPs were in LD and what the extent of LD was among them. LD analysis revealed that rs7694620 and rs3087494 were tightly linked and rs3087494 and rs2285714 were tightly linked. The frequency distributions of the rs7694620-rs3087494 and rs3087494-rs2285714 haplotype systems in *PLA2G12A* were significantly different between cases and controls (*P*<0.001). This finding suggested a significant association of two adjacent haplotype blocks in *PLA2G12A* with schizophrenia. In addition, both allelic and genotypic association analyses showed a significant association between SNP rs3087494 and schizophrenia. One interesting finding in our study was the interaction effect of rs3087494 and gender. The SNP rs3087494 had a negative interaction with gender. This interaction may weaken the effect of rs3087494 association with schizophrenia.

Our previous findings on the relationship between PLA2 gene family SNPs and schizophrenia suggest that the BanⅠSNP in *PLA2G4A* was associated with symptoms of schizophrenia but the *PLA2G4A* SNP rs7542180 was not associated with schizophrenia [24]. SNP rs1648833 in *PLA2G4B* was associated with schizophrenia in males but SNP rs3816533 in *PLA2G4B* was not associated with schizophrenia [25]. SNP rs1549637 in *PLA2G4C* was also found to be associated with schizophrenia, but the positive results failed to be replicated in other case-control studies [26]. SNPs rs4924618 and rs2459692 in *PLA2G4D* were not significantly associated with schizophrenia. Negative results were obtained from SNP rs4924595 in PLA2G4E as well. In addition, the results from other studies were also inconsistent. Genetic studies suggested that the PLA2G4A gene, coding for a cytosolic form of PLA2, may be associated with schizophrenia [13, 27], although failure to replicate the *PLA2G4A* SNP association results has been reported [16, 19, 28]. The results from a case-control study in South Korea were in line with the results of previous studies that showed a positive association of PLA2 alleles with schizophrenia [13].

There are several limitations in the present study that should be considered. First, because this is a cross-sectional study, the interpretation of causal relationships between risk factors and schizophrenia is limited. Second, our data were obtained from a cross-sectional study in the Jilin Province and were not representative of adults throughout China. Furthermore, our study was performed at a single center, which may ignore other SNPs in the candidate genes associated with schizophrenia. At the same time, when we collected samples, we did not record enough quantitative traits. Therefore, we have not observed whether the significant SNPs have eQTL effects or are linked to coding variants. Despite these limitations, our current study provides a sufficient sample size to detect this psychiatric marker. More functional genomics and pathogenesis should be performed in future studies.

## Conclusions

The genetic association of these genes with schizophrenia should be confirmed further and could be particularly vital for the understanding of the relationship between schizophrenia and PLA2. There is no doubt that more investigations of genetic associations of genes with schizophrenia would be substantially important for the analysis of the membrane phospholipid hypothesis of schizophrenia.
